# Distinct Factors Shape Aquatic and Sedimentary Microbial Community Structures in the Lakes of Western China

**DOI:** 10.3389/fmicb.2016.01782

**Published:** 2016-11-08

**Authors:** Jian Yang, Hongchen Jiang, Geng Wu, Wen Liu, Guojing Zhang

**Affiliations:** State Key Laboratory of Biogeology and Environmental Geology, China University of GeosciencesWuhan, China

**Keywords:** lakes, biogeography, aquatic microbial community, sedimentary microbial community, environmental factors, spatial factors

## Abstract

Little is known about the relative importance of spatial and environmental factors to structuring aquatic and sedimentary microbial biogeography in lakes. Here, we investigated the microbial community composition (MCC) of the water (*n* = 35) and sediment (*n* = 35) samples from 16 lakes in western China (salinity: freshwater to salt saturation; pairwise geographical distance: 9–2027 km) using high-throughput sequencing and evaluated the relative importance of spatial and environmental factors to microbial (including total, abundant, and rare) distributions. Our results showed that spatial factors were more important than environmental factors in shaping the biogeography of aquatic and sedimentary microbial communities in the studied lakes, and spatial factors on abundant microbial community was stronger than that on the total/rare microbial communities. Moreover, sedimentary rare MCC might be more sensitive to environmental factors than its aquatic counterpart. Such different biogeography responses of total, abundant, and rare communities to environmental and spatial factors could be ascribed to different physiochemical properties between water and sediment. Collectively, this study expands our understanding of factors shaping microbial biogeography of total, abundant, and rare communities between waters and sediments of lakes.

## Introduction

Microbes play central roles in regulating elemental cycles of carbon, nitrogen, and sulfur within lakes (Newton et al., 2011; Sorokin et al., 2014). Microbial functions involved in carbon, nitrogen, and sulfur cycling are commonly related to microbial community composition (MCC; Graham et al., 2014, 2016). MCC varies among lakes with different environmental variables. Thus, studies on MCC of lakes and their biogeographical patterns are of great importance to understanding of microbial functions in lacustrine ecosystems (Van der Gucht et al., 2007).

Lake water and sediment are different habitats, each with unique intrinsic environmental conditions (e.g., redox potential; Jiang et al., 2006; Feng et al., 2009) and MCCs (DeLong et al., 1993; Jiang et al., 2006; Mesbah et al., 2007; Yang et al., 2013). Such difference may account for different microbial biogeography in lakes (Lindström and Langenheder, 2012). Up to now, a great number of studies have investigated the relative influence of environmental and spatial factors on microbial biogeography in lakes (Beisner et al., 2006; Langenheder and Ragnarsson, 2007; Van der Gucht et al., 2007; Pagaling et al., 2009; Schiaffino et al., 2011; Xiong et al., 2012; Logares et al., 2013; Souffreau et al., 2015). Most previous studies found that environmental factors were more important than spatial factors in influencing aquatic and sedimentary MCCs (Van der Gucht et al., 2007; Xiong et al., 2012; Logares et al., 2013; Souffreau et al., 2015); while others showed that spatial factors (e.g., geographic distance) also impacted aquatic and sedimentary MCCs (Beisner et al., 2006; Langenheder and Ragnarsson, 2007; Pagaling et al., 2009; Schiaffino et al., 2011; Xiong et al., 2012). However, most of these previous studies separately investigated either aquatic or sedimentary MCC among multiple lakes. Thus little is known about the difference of the relative importance of environmental and spatial factors to the distributions of aquatic and sedimentary microbial MCCs in lakes at large spatial scales (e.g., tens to thousands of kilometers).

In addition, in natural environments, microbial communities within one ecosystem could be classified into abundant and rare taxa with respect to their biomass and biodiversity contributions, with the former contributing major biomass but minor biodiversity while the latter contributing minor biomass but major biodiversity to ecosystems (Pedrós-Alió,, 2012; Logares et al., 2014). Previous studies show that rare taxa may conduct more crucial ecosystem functions than their abundant counterparts (Pester et al., 2010; Lawson et al., 2015). Considering different contributions of abundant and rare taxa to biomass and biodiversity, different factors may account for their distinct biogeography (Pedrós-Alió,, 2012; Liu et al., 2015). However, such knowledge is limited. So it is essential to discern the distinct distribution patterns of abundant and rare taxa and their respective influencing factors in natural environments.

The biogeography of microbial abundant and rare taxa in waters of freshwater lakes have been investigated. For example, one study indicated that the distribution of rare taxa was mostly influenced by local environmental factors (e.g., electrical conductivity), whereas that of abundant taxa was predominately affected by spatial factors (Liu et al., 2015). Another study suggested that local environmental factors (e.g., salinity) influenced the distribution of both abundant and rare microbial taxa in waters of a number of coastal lakes with a range of salinity (0–100 g/L; Logares et al., 2013). Such inconsistency could be caused by different environmental factors (e.g., salinity) among those investigated lakes. However, it is still unclear how differently environmental and spatial factors influence the distribution of abundant and rare microbial taxa in water and sediment of lakes as a function of salinity (e.g., from freshwater to up to salt saturation).

The objective of this study was to examine the relative importance of environmental and spatial factors to structuring the distribution of microbial communities (including total, abundant, and rare communities) in water and sediment of lakes with a wide range of salinity from freshwater to up to salt saturation. In this study, the water (*n* = 35) and sediment (*n* = 35) samples from a total of 16 lakes in Tibet (*n* = 7), Qinghai (*n* = 6), and Xinjiang (*n* = 3) Provinces, western China were investigated by using high-throughput sequencing of 16S rRNA gene amplicons.

## Materials and Methods

### Sample Collection

In 2014 summer, surface (∼0–5 cm) water and surface (∼0–5 cm) sediment samples were collected from inshore sites with water depth of ∼1.0 meters in 16 lakes of western China (Supplementary Table S1). The data of mean annual temperature (MAT) and mean annual precipitation (MAP) was obtained from local weather stations where the lakes are situated. The pH of these lakes was measured with a portable pH meter (PT-10, Sartorius, Germany) in the field. Surface waters were collected using 2-L autoclaved polycarbonate bottles (Nalgene, USA). Aquatic biomass in surface water was collected by filtering ∼500 mL water through 0.2-μm Isopore filters (Whatman, UK). The filtrate (∼40 mL) of lake water was collected into 50-mL sterilized Teflon tubes for subsequent major ions concentration analyses in laboratory. Water samples for dissolved organic carbon (DOC) analysis were collected by filtrating surface water through 0.7-μm Whatman GF/F filters followed by acidification with concentrated phosphoric acid. The resulted DOC samples were stored in ice in the field and during transportation and were stored at 4°C in laboratory. Surface sediments were collected with a grab-bucket collection sampler and then put into 15-mL sterilized tubes using sterile spoons for DNA extractions and total organic carbon (TOC) measurements. All water and sediment samples for DNA extractions were stored on dry ice in the field and during transportation, and were stored at -80°C in laboratory until further analyses.

### Laboratory Geochemical Analyses

Cation and anion concentrations (e.g., K^+^, Na^+^, Ca^2+^, Mg^2+^, ${\mathrm{SO}}_{4}^{2–}$, Cl^-^, ${\mathrm{NO}}_{2}^{–}$, ${\mathrm{NO}}_{3}^{–}$) of the lake waters and sediment pore waters were measured by using ion chromatography (Dionex DX-600, USA). Salinity was obtained by summing the concentration of six major ions including K^+^, Na^+^, Ca^2+^, Mg^2+^, ${\mathrm{SO}}_{4}^{2–}$, and Cl^-^. Water DOC and sediment TOC concentrations were measured on a multi N/C 2100S analyzer (Analytik Jena, Germany). Before sediment TOC analysis, samples were firstly acidified with 1 N HCl overnight to remove carbonates, subsequently washed to neutral pH, dried in an oven and ground into fine powder.

### DNA Extraction and Sequencing

DNA was extracted from biomass-containing filters and 0.5 g sediment samples using the Fast DNA SPIN Kit for Soil (MP Biomedical, Solon, OH, USA). The extracted DNA was amplified with a universal 16S rRNA gene primer set of 515F (5′-GTGYCAGCMGCCGCGGTA-3′)/909R (5′-CCCCGYCAATTCMTTTRAGT-3′), and the detailed PCR conditions were described in a previous study (Tamaki et al., 2011). Briefly, a unique 12 bp barcode sequence was added between the sequencing adapter and forward primer to differentiate among samples. Triplicate PCRs for each sample were conducted and purified using a DNA Gel Extraction Kit (Axygen, Union City, CA, USA). The bar-coded amplicons from each sample were pooled with equimolar concentrations and then were sequenced by using an Illumina Miseq platform (Caporaso et al., 2012).

### Data Processing and Statistical Analyses

The raw data were processed using the QIIME v1.8.0 (Caporaso et al., 2010). The paired reads were joined with FLASH (fast length adjustment of short reads) using default setting (Magoè and Salzberg, 2011). Chimera checking was performed using the UCHIME module of the USEARCH program (Edgar et al., 2011). Operational taxonomic units (OTUs) were defined at the 97% cutoff by using the UCLUST algorithm (Edgar, 2010). OTU representative sequences were selected and their taxonomy were assigned using the ribosomal database project (RDP) classifier algorithm at the 80% threshold (Wang et al., 2007). The OTUs each comprising only one sequence were removed prior to further analysis to minimize sequencing artifacts. The OTU table was rarefied to equal sequence number (*n* = 1691) for each sample with 1000 times, and then alpha diversity (i.e., Simpson, Shannon, Equitability, and Chao1) was calculated by QIIME at the 97% identity level. The rarefied OTU table was used for downstream analysis, unless otherwise specified. In addition to analysis of different fractions of a community and their response to environmental factors, we separately classified abundant and rare taxa based on OTU relative abundance within each sample. Specifically, abundant and rare OTUs were arbitrarily defined as the OTUs with relative abundance of >1 and <0.1% within one sample, respectively (Pedrós-Alió,, 2012). The rare taxa were defined by the criteria of <0.1% in this study, because the minimum number of sequences among our samples was 1691 and the 0.1% threshold can give the almost lowest frequency OTUs that were represented by only several (<2) reads in our samples.

All statistical analyses were carried out in the R program^1^ implemented with various packages unless otherwise indicated. In order to assess the difference of MCC among sample groups of different locations (i.e., Tibet, Qinghai, and Xinjiang Provinces) and sample types (i.e., water vs. sediment), analysis of similarities (ANOSIM) was performed based on Bray–Curtis dissimilarity with 9999 permutations using R package “vegan.” SIMPER (similarity percentage) analysis was conducted to rank the taxa that contributed to the differences among sample groups described above using the PAST software^2^. The mean abundances of those top ranked taxa in each group were also calculated in the SIMPER analysis.

The normality of the environmental variables was checked using Shapiro–Wilk test and all variables in this study were normalized to values ranged between 1 and 100 as described previously (Liu et al., 2014). In order to accurately predict and explain the relationships between ecological data and environmental variables, aggregated boosted tree (ABT) analysis (with 5000 trees used for the boosting, 10-folds cross-validation, and three-way interactions) was performed to quantitatively evaluate the relative influence of individual environmental factors and geographic distance on the distribution of microbial community based on Bray–Curtis dissimilarity using R package “gbm” (De’ath, 2007). The ABT analysis can give relative influence of individual environmental parameters on MCC, but it is limited to quantitatively assess how much MCC variations can be separately explained by environmental factors and spatial factors. In order to quantify the relative importance of environmental and spatial factors in shaping microbial community, a canonical correspondence analysis (CCA)-based variation partitioning analysis (VPA) was carried out according to the methods described previously (Jiang et al., 2014). Briefly, a set of spatial variables were firstly produced through the method of principal coordinates of neighbor matrices (PCNM) analysis according to the longitude and latitude coordinates of sampling sites (Borcard and Legendre, 2002). Subsequently, variance inflation factors (VIFs) were computed to check the presence of collinearities among environmental variables using the function *vif.cca* in the “vegan” package. If the maximum VIF was more than 10, the environmental variables which had the smallest relative influence (results from ABT analysis) were removed until all VIFs of variables were stay <10. Finally, only significant (*P* < 0.05) environmental and spatial variables that were tested by CCA with 1,000 permutations were kept for variance partition analysis (VPA) using the “vegan” package.

### Nucleotide Sequence Accession Numbers

The original sequences were deposited at the Sequence Read Archive (SRA) in the National Center for Biotechnology Information (NCBI) under the BioProject accession no. SRP056907.

## Results

### Environmental Parameters of the Sampled Lakes

The geographic locations and environmental parameters of the studied lakes were summarized in Supplementary Figure S1 and Supplementary Table S1. Briefly, the salinity of the sampled lakes ranged from 0.1 to 354.1 g/L, and pH varied from 6.9 to 9.8. MAT and MAP of the lake regions were -1.2–9.1°C and 70.0–456.8 mm, respectively. Pairwise distances between the sampled lakes ranged from 9 to 2027 km.

### Composition and Diversity of Lake Microbial Communities

In total, 390,052, and 352,672 quality sequence reads were obtained from water (*n* = 35) and sediment (*n* = 35) samples with an average of 11,444 and 10,076 sequence reads per sample, respectively. Alpha diversity indices of water and sediment samples were summarized in Supplementary Tables S2A,B. Briefly, the observed OTUs were 240.6–584.3 and 326.5–701.5 for water and sediment samples, respectively; the Shannon indices were 4.3–8.0 and 6.1–8.6 for water and sediment samples, respectively; the Chao 1 indices were 440.2–1481.0 and 846.0–1790.7 for water and sediment samples, respectively (Supplementary Tables S2A,B). Across all the studied lake water samples, the dominant phyla (average relative abundances >1%) were *Actinobacteria, Bacteroidetes, Cyanobacteria, Euryarchaeota, Planctomycetes, Proteobacteria*, and *Verrucomicrobia*; whereas the dominant phyla of the studied lake sediment samples were *Acidobacteria, Actinobacteria, Bacteroidetes, Chloroflexi, Crenarchaeota, Cyanobacteria, Euryarchaeota, Firmicutes, Planctomycetes, Proteobacteria, Spirochaetes*, and *Thermi*. Furthermore, ANOSIM also indicated that MCCs between lake water and sediment samples were significantly (*R* = 0.403, *P* < 0.001) distinct.

Among the water samples, a total of 10–20 OTUs were classified as abundant OTUs. These abundant OTUs accounted for 2.4–8.6% of total OTUs and represented 33.6–78.5% relative abundance within each corresponding sample; a total of 106–396 rare OTUs were identified and they accounted for 48.4–80.9% of total OTUs and 6.3–23.4% relative abundance within each corresponding sample (Supplementary Table S3A). In addition, among the sediment samples a total of 6–23 OTUs were classified as abundant OTUs, which accounted for 0.7–5.7% of total OTUs and represented 15.7–67.6% of relative abundance in each corresponding sample; whereas a total of 206–564 rare OTUs were identified and they accounted for 61.2–73.7% of total OTUs and 12.2–33.4% of relative abundance in each corresponding sample (Supplementary Table S3B).

### Geographical Patterns of Microbial Community

Geographical distribution patterns were observed for the total microbial communities of lake water and sediment samples in this study. ANOSIM indicated that significant (*P* < 0.05) total MCC dissimilarities were found among the sampling locations (Tibet, Qinghai, and Xinjiang Provinces; **Table 1**). SIMPER analyses showed that the overall average dissimilarities were 53.6 and 51.3% for the aquatic and sedimentary microbial communities across different locations, respectively. Moreover, a total of 15 and 22 major classes contributed (with each contributing >1%) to the observed dissimilarities between aquatic and sedimentary microbial communities across the three sampling areas, and the composition of those classes were distinct between waters and sediments in the studied lakes (Supplementary Table S4), among which the *Actinobacteria* and *Betaproteobacteria* showed the most contribution (12.1 and 14.7%) to the aquatic and sedimentary microbial community dissimilarities in the studied samples, respectively (Supplementary Table S4). In addition, ANOSIM further indicated that abundant MCCs significantly (*P* < 0.05) differed among the three sampling locations (Tibet, Qinghai and, Xinjiang Provinces), whereas rare taxa compositions did not show significant difference among locations (Supplementary Table S5).

**Table 1:** Relative influence of individual environmental parameters and geographic distance on MCCs in the waters and sediments of the sampled lakes from Tibet, Qinghai and Xinjiang Provinces, western China.

	Water			Sediment		
	All	Abundant	Rare	All	Abundant	Rare
SAL	34.0	20.0	32.5	45.2	19.0	39.3
GD	46.0	55.4	14.8	33.2	43.3	18.6
pH	1.3	1.2	3.9	1.4	2.0	3.3
DOC/TOC	1.0	1.4	23.4	1.5	3.1	4.8
MAP	7.6	9.9	4.1	2.6	15.2	7.1
MAT	5.8	6.7	10.2	11.0	6.9	10.5
NO_2_	0.6	0.9	7.2	2.6	3.3	13.2
NO_3_	3.7	4.5	3.9	2.5	7.2	3.1

*SAL, salinity; GD, geographic distance; MAT, mean annual temperature; MAP, mean annual precipitation; DOC, dissolved organic carbon in lake water; TOC, total organic carbon in lake sediment.*

### Relative Influence of Individual Environmental Parameters and Geographic Distance on Microbial Distribution

The ABT analysis showed that salinity and geographic distance had highest relative influence (45.2 and 46.0%) on the total sedimentary and aquatic MCCs, respectively. However, geographic distance possessed highest relative influence (55.4 and 43.3%) on the aquatic and sedimentary abundant MCCs, which is much higher than that (14.8 and 18.6%, respectively) on the aquatic and sedimentary rare MCCs (**Table 1**).

### Relative Importance of Environmental and Spatial Factors on Microbial Distribution

**FIGURE 1:**
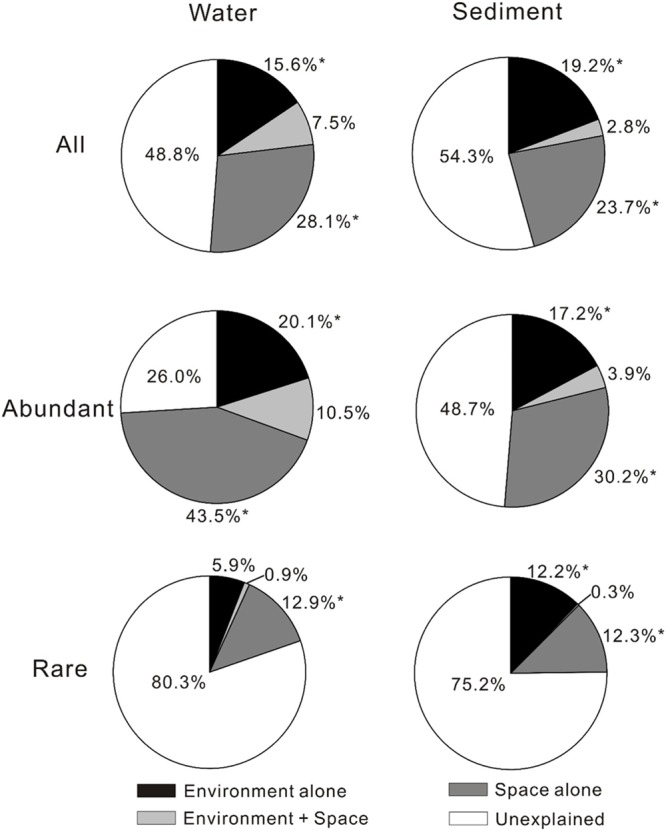
**Variance partition analysis showing relative importance of environmental and spatial factors in shaping microbial community compositions (MCCs) in the lake waters and sediments of the studied lakes (The significances were tested by canonical correspondence analysis (CCA) with 1000 permutations).** ^∗^*P* < 0.05.

The VPA results showed that spatial factors exhibited higher contribution to the MCC variations than environmental factors although the relative contributions of spatial and environmental factors to the MCC variations differed with respect to total, abundant, and rare microbial communities (**Figure 1**). For total microbial community, spatial factors alone gave much higher (28.1% vs. 15.6% for waters and 23.7% vs. 19.2% for sediments, respectively) explanation on the MCC variation than the environmental factors alone in the studied lakes. For the abundant sub-community, spatial factors alone also had much higher explaining power on the variation of both aquatic and sedimentary microbial communities than the environmental factors. For the rare sub-communities, spatial factors alone presented much higher (12.9% vs. 5.9%) explanation on the variation of aquatic microbial community than the environmental factors alone, while the environmental and spatial factors alone did not exhibited much different (12.2% vs. 12.3%) explanation on the variation of sedimentary microbial community. Interestingly, the explaining power of spatial factors on the variation of the aquatic microbial community (including total, abundant, and rare community) were also higher than that of the sedimentary microbial community.

## Discussion

### Relative Importance of Environmental and Spatial Factors in Influencing Total Aquatic and Sedimentary MCCs

Between the two types of influencing factors (spatial vs. environmental), spatial factors might be more important than environmental factors in shaping the biogeography of the aquatic and sedimentary total microbial communities in the studied lakes. This finding was inconsistent with previous reports in that environmental rather than spatial factors strongly affect microbial biogeography in lakes at a large distance scale (>2000 km; Van der Gucht et al., 2007; Schiaffino et al., 2011; Souffreau et al., 2015). This inconsistency may be ascribed to two possible reasons: one is that different fingerprinting methods were employed: the high-throughput sequencing technique (Illumina Miseq) in the present study vs. denaturing gradient gel electrophoresis (DGGE) in those previous studies. The DGGE technique has intrinsic limitations regarding its low resolution: it tends to detect microbial species with relative abundance of >0.1% but fails for rare microbes (Galand et al., 2009; Van Elsas and Boersma, 2011; Zhi et al., 2014). Therefore, DGGE may be limited for understanding the diversity (especially rare taxa) and biogeography of the whole microbial community in natural environments. In contrast, high-throughput sequencing (e.g., Illumina Miseq) can provide more sequence reads and higher sequencing depth than traditional methods (e.g., DGGE, cloning library-based sequencing), and thus it is more suitable for understanding microbial biogeography (Pedrós-Alió,, 2012; Logares et al., 2014; Liu et al., 2015). So it is not surprising to observe the different relative importance of spatial and environmental factors on shaping microbial biogeography between this and those studies; the other possible reason is that other unmeasured environmental variables (e.g., total nitrogen, total phosphorus, chlorophyll-a, bacterial predators) might contribute to the different MCCs among the studied lakes. Some of the unmeasured variables have shown great effect on the distribution of lake microbial communities in previous studies. For example, Souffreau et al. (2015) found that bacterial predators (e.g., cladocerans), total phosphorus, and nitric nitrogen significantly influenced MCCs of lakes. In addition, VPA further validated that a large proportion of the observed MCC variations could not be explained by the measured environmental and spatial variables (**Figure 1**). Therefore, the relative importance of spatial and environmental variables on microbial distribution awaits further investigation.

It is notable that spatial factors have stronger influence on the variation of the aquatic microbial community (including total, abundant, and rare community) than that of its sedimentary counterpart. Such different influence of spatial factors on aquatic and sedimentary microbial communities may be due to the different physicochemical conditions between water and sediment. Microbes can be easily spread from place to place (even thousands of kilometers away) by attaching to dusts, aerosols, animals, and other solid particles (Hughes et al., 2004; Green and Bohannan, 2006; Crump et al., 2012; Wilkinson et al., 2012; Barberán et al., 2015). Therefore, it is highly possible for microbes to immigrate among lakes. The environmental selection may influence microbial biogeography between water and sediment among the lakes: lake waters are the first habitat for exotic microbes to adapt, and the arriving microbes are firstly selected by water physicochemical conditions. Some of the surviving exotic microbes would reach lake sediments, and were then subjected to another selection by sedimentary physicochemical conditions. Thus the effect of spatial factors (e.g., geographic distance) on sedimentary MCC was weaken by the double environmental selections. So it is reasonable to observe that the spatial effect on the aquatic microbial community was much stronger than that on its sedimentary counterpart (**Figure 1**)

### Relative Importance of Environmental and Spatial Factors on the Distributions of Abundant and Rare Microbes

It is remarkable that spatial factors were more important than environmental factors in shaping the biogeography of aquatic and sedimentary abundant microbial communities, and the spatial effect on the variation of abundant microbial community was stronger than that of its total and rare counterparts in both water and sediment of the lakes. This observation was consistent with a previous study in freshwater lakes of eastern China (Liu et al., 2015), but was not in agreement with another study (Logares et al., 2013) in coastal lakes with a large salinity gradient (0–100 g/L) that is similar to this study (0.1–354.1 g/L). Logares et al. (2013) suggested abundant MCC was mainly affected by environmental factors when environmental filtering (e.g., salinity) was strong. This discrepancy may be ascribed to the spatial distance differences among the studied lakes and/or to the methodology between the present and that studies (Logares et al., 2013). For example, our sampled lakes are all inland lakes with a distance range of up to 2000 km, much larger than that (∼20 km) for coastal lakes in that study (Logares et al., 2013). Moreover, the abundant OTUs in the present study were defined as those with >1% relative abundance within one sample, in contrast with those containing >100 reads per sample in that study (Logares et al., 2013). The definition of abundant taxa in this study has been widely employed in many previous studies (Galand et al., 2009; Pedrós-Alió,, 2012; Logares et al., 2014; Liu et al., 2015), and thus it may be more comparable than that in Logares et al. (2013). In addition, it may be reasonable to observe that spatial factors influenced the distribution of abundant microbes more significantly than that of total and rare counterparts in lake waters and sediments. Because abundant microbes can utilize a wide spectrum of substrates and easily reach high abundance when they arrive in a new habitat (Hambright et al., 2015), and thus they may have high probability of dispersal and strong immigrating capability (Liu et al., 2015).

It is also noticeable that environmental and spatial factors were of different importance in shaping the biogeography of the rare microbial communities between waters and sediments of the lakes. Our results showed that the variation of the aquatic rare MCC was significantly explained by spatial rather than environmental factors, which was not in accordance with previous studies (Logares et al., 2013; Liu et al., 2015). In contrast, the sedimentary rare MCC was significantly explained by both environmental and spatial factors with each showing almost equal explaining power, suggesting that sedimentary rare MCC might be more sensitive to environmental factors than their aquatic counterparts. Such inconsistency for the biogeography between aquatic and sedimentary rare microbial communities may be ascribed to reasons similar to that for total microbial community in that physicochemical difference between water and sediment resulted in the distinct microbial biogeography.

## Conclusion

Spatial factors were more important than environmental factors in affecting the distributions of aquatic and sedimentary MCCs in the studied lakes, and the spatial effect on abundant microbial community was stronger than that on its total and rare counterparts in both waters and sediments of the lakes. Furthermore, sedimentary rare MCC might be more sensitive to environmental factors than its aquatic counterpart. Such differences in spatial and environmental effects on microbial biogeography to could be ascribed to different physicochemical properties between water and sediment of the lakes. In addition, some unmeasured variables may also influence the microbial biogeography in lakes, which awaits further investigation.

## Author Contributions

HJ and JY conceived and designed the experiments; JY, GW, WL, and GZ performed the experiments; JY analyzed the data. All of the authors assisted in writing the manuscript, discussed the results and commented on the manuscript.

## Conflict of Interest Statement

The authors declare that the research was conducted in the absence of any commercial or financial relationships that could be construed as a potential conflict of interest.

## Footnotes

1 http://cran.r-project.org/

2 http://folk.uio.no/ohammer/past/
