# Supplementary material for: Distinct Factors Shape Aquatic and Sedimentary Microbial Community Structures in the Lakes of Western China
Source: Front Microbiol. 2016 Nov 8;7:1782. doi: 10.3389/fmicb.2016.01782 (PMC5099527; doi:10.3389/fmicb.2016.01782)
Supplement: Supplementary file 1 [file Data_Sheet_1.pdf]

Distinct factors shape aquatic and sedimentary microbial community structures in the lakes of western China

Running title: Biogeography of lacustrine microbes

Jian Yang, Hongchen Jiang*, Geng Wu, Wen Liu and Guojing Zhang

State Key Laboratory of Biogeology and Environmental Geology, China University of Geosciences, Wuhan, 430074, China

*Corresponding authors:

Hongchen Jiang: jiangh@cug.edu.cn; Tel: 86-27-67883452

Revised for Frontiers in Microbiology

Oct 4th, 2016

Supplementary information

Table S1 GPS Locations and physicochemical parameters of samples in the studied lakes.

Table S2 Alpha-diversity of the water (A) and sediment (B) in the studied lakes.

Table S3 Distribution and relative abundance of the abundant and rare OTUs of the water (A) and sediment (B) in the studied lakes.

Table S4 SIMPER analyses identify the top classes that contributed at least 1% to the dissimilarity between the microbial communities of three sampling locations.

Table S5 Dissimilarity tests of the total, abundant and rare communities among the samples of different locations (i.e. Tibet, Qinghai and Xinjiang Provinces).

Fig. S1 A geographic map showing the locations of the sampled lakes in western China.

Table S1 GPS Locations and physicochemical parameters of samples in the studied lakes (MAT: mean annual temperature; MAP: mean annual precipitation; TOC: total organic carbon; DOC: dissolved organic carbon. * The asterisk indicates that TOC and DOC data were derived from sediment and water samples, respectively).

Sample type	Area	Lake name	Sample ID	GPS Location	MAT (ºC)	MAP (mm)	Salinity （g/L）	pH	*TOC(%) /DOC(mg/L)	Na^+ (mg/L)	K^+ (mg/L)	Mg^{2+} (mg/L)	Ca^{2+} (mg/L)	Cl^- (mg/L)	SO_4^{2-} (mg/L)	NO_2^- (mg/L)	NO_3^- (mg/L)
Lake Water	Qinghai	Qinghai Lake	QWA1	36º33'15.8"N/100º37'30.2"E	1.2	336.6	11.5	9.2	38.5	3419.5	136.0	676.0	0.0	5295.4	1959.7	56.3	24.2
		Qinghai Lake	QWA2	36º33'12.3"N/100º37'41.1"E	1.2	336.6	11.7	9.2	20.3	3633.0	144.9	716.2	0.0	5255.3	1947.9	56.9	19.2
		Erhai Lake	QWB1	36º33'22.6"N/100º43'17.8"E	1.2	336.6	0.7	9.3	1.5	237.4	12.1	79.9	12.3	262.6	118.2	14.0	2.5
		Erhai Lake	QWB2	36º33'22.6"N/100º43'17.8"E	1.2	336.6	0.7	9.4	1.3	244.3	12.1	80.7	11.1	259.2	135.1	13.0	3.2
		Erhai Lake	QWB3	36º33'22.6"N/100º43'17.8"E	1.2	336.6	0.8	9.3	534.6	250.2	12.1	80.5	12.9	255.5	170.0	14.6	2.3
		Gahai Lake1	QWC1	36º43'50.4"N/100º40'19.2"E	1.5	400.0	29.7	8.6	1.7	9624.5	487.7	1383.3	0.0	12934.0	5308.7	0.0	185.0
		Gahai Lake1	QWC2	36º43'50.4"N/100º40'19.2"E	1.5	400.0	30.4	8.3	2.8	9920.4	486.6	1435.3	0.0	13037.8	5531.8	43.0	261.8
		Gahai Lake1	QWC3	36º43'50.4"N/100º40'19.2"E	1.5	400.0	30.2	8.7	72.2	9716.5	568.7	1401.0	0.0	13115.5	5387.2	0.0	181.0
		Chaka Lake	QWD1	36º45'01.4"N/99º04'48.1"E	3.5	159.3	347.5	7.3	222.7	65729.9	6517.1	39889.6	211.1	196176.8	38970.7	732.2	492.1
		Chaka Lake	QWD2	36º45'01.4"N/99º04'48.1"E	3.5	159.3	345.4	6.9	29.2	65460.2	6541.7	40054.3	198.3	194537.0	38631.5	671.7	271.2

		Chaka Lake	QWD3	36º45'01.4"N/99º04'48.1"E	3.5	159.3	354.1	6.9	24.1	66767.1	6662.1	40883.7	209.9	200196.1	39368.9	513.5	256.6
		Gahai Lake2	QWE1	37º08'10.8"N/97º34'38.2"E	3.0	100.0	81.9	8.2	19.8	25519.9	397.6	3774.2	59.1	41439.8	10662.9	83.1	257.2
		Gahai Lake2	QWE2	37º08'10.8"N/97º34'38.2"E	3.0	100.0	77.6	8.3	22.1	24361.2	393.9	3586.8	27.9	39286.0	9955.7	60.2	191.1
		Gahai Lake2	QWE3	37º08'10.8"N/97º34'38.2"E	3.0	100.0	81.2	8.3	51.6	25629.7	409.9	3752.2	35.1	41015.4	10383.0	52.6	193.8
		Xiaochaidan Lake	QWF1	37º27'09.2"N/95º30'38.1"E	1.4	83.5	35.8	8.4	1.7	12824.6	221.7	731.3	0.0	16159.2	5907.2	0.0	275.7
		Xiaochaidan Lake	QWF2	37º27'09.2"N/95º30'38.1"E	1.4	83.5	36.4	8.5	25.8	12863.0	213.6	758.8	0.0	16620.8	5988.5	0.0	216.1
		Xiaochaidan Lake	QWF3	37º27'09.2"N/95º30'38.1"E	1.4	83.5	128.8	8.1	481.6	44197.0	730.4	2707.3	233.2	56704.7	24221.7	124.3	225.8
		Tuosu Lake	QWG1	37º11'38.9"N/96º53'19.4"E	3.0	100.0	28.1	8.9	1.4	8135.7	218.4	2056.7	0.0	11910.2	5763.5	16.4	239.2
		Tuosu Lake	QWG2	37º11'38.9"N/96º53'19.4"E	3.0	100.0	28.4	8.9	52.0	8428.8	298.7	2008.5	0.0	11977.8	5677.9	0.0	194.1
		Tuosu Lake	QWG3	37º11'38.9"N/96º53'19.4"E	3.0	100.0	27.4	9.0	78.8	8022.6	219.4	2018.1	0.0	11601.7	5542.2	0.0	218.0
	**Tibet**	Angrenjincuo	TWA	29º18'30.1"N/87º13'10.8"E	6.5	400.0	0.2	9.8	80.8	70.2	3.0	10.5	7.6	21.0	45.9	0.0	40.6
		Langcuo	TWB	29º12'44.1"N/87º22'58.8"E	6.5	400.0	4.0	9.6	92.0	2706.9	7.2	25.6	0.0	253.7	961.6	139.2	38.9
		Jiadamangcuo	TWC	29°36'26.7"N/85°44'55.2"E	4.0	300.0	0.1	7.3	386.5	13.7	1.7	1.6	13.4	7.2	17.1	3.0	6.1

		Namucuo	TWG1	30°52'17.3"N/91°02'40.9"E	1.3	456.8	0.6	9.1	59.7	239.7	27.2	91.7	0.0	73.7	192.6	25.1	36.3
		Namucuo	TWG2	30°52'17.3"N/91°02'40.9"E	1.3	456.8	0.6	9.0	79.3	237.8	27.6	86.5	0.0	73.8	195.3	24.1	36.2
		Namucuo	TWG3	30°52'17.3"N/91°02'40.9"E	1.3	456.8	0.6	9.1	435.9	242.6	27.7	88.2	0.0	72.3	184.5	23.5	35.6
		Yangzhuo yongcuo	TWH1	29°07'36.1"N/90°27'0.4"E	3.8	353.2	1.0	8.8	83.8	230.0	23.8	167.8	0.0	33.0	527.4	16.0	31.2
		Yangzhuo yongcuo	TWH2	29°07'36.1"N/90°27'0.4"E	3.8	353.2	1.0	8.8	84.7	224.0	19.9	162.9	0.0	35.5	528.9	14.4	33.2
		Yangzhuo yongcuo	TWH3	29°07'36.1"N/90°27'0.4"E	3.8	353.2	1.0	8.8	47.1	218.6	19.2	160.5	0.0	35.3	556.7	18.2	32.2
		Bangecuo	TWK	31°45'05.1"N/89°30'20.1"E	0.0	340.5	60.1	9.5	112.8	20925.6	2363.1	192.7	0.0	12874.6	23728.4	199.7	38.1
	**Xinjiang**	Wulungu Lake	XWA1	47º13'18.1"N/87º22'32.1"E	4.7	131.0	2.3	8.6	100.2	356.9	48.3	59.2	0.0	759.3	1039.0	0.0	0.0
		Wulungu Lake	XWA2	47º13'14.8"N/87º22'29.2"E	4.7	131.0	2.1	8.5	1.4	338.5	39.5	46.2	0.0	577.8	1057.6	0.0	403.1
		Wulungu Lake	XWA3	47º13'16.8"N/87º22'26.4"E	4.7	131.0	2.1	8.6	72.7	333.1	38.8	51.8	0.0	585.4	1084.0	0.0	401.7
		Sailimu Lake	XWB1	44º33'13.7"N/81º20'37.4"E	1.1	400.0	2.1	8.8	22.6	288.3	21.6	387.3	0.0	307.4	1051.8	14.5	55.5
		Chaiwobu Lake	XWE1	43º30'29.5"N/87º56'09.3"E	6.0	70.0	3.7	7.8	90.9	1028.5	7.0	54.1	0.0	792.0	1831.6	0.0	436.5
	**Qinghai**	Qinghai Lake	QSEDA1	36º33'15.8"N/100º37'30.2"E	1.2	336.6	7.9	9.2	1.5	2493.0	200.3	2152.3	357.7	2454.9	217.9	494.9	51.5

**Lake sediment**		Qinghai Lake	QSEDA2	36º33'15.8"N/100º37'30.2"E	1.2	336.6	9.6	9.2	2.2	2736.9	229.6	2902.1	474.8	2730.1	547.9	599.0	49.7
		Erhai Lake	QSEDB1	36º33'22.6"N/100º43'17.8"E	1.2	336.6	0.7	9.3	0.7	237.4	12.1	79.9	12.3	262.6	118.2	14.0	2.5
		Erhai Lake	QSEDB2	36º33'22.6"N/100º43'17.8"E	1.2	336.6	0.9	9.4	1.2	265.4	14.6	77.1	0.0	344.5	190.5	9.2	5.0
		Erhai Lake	QSEDB3	36º33'22.6"N/100º43'17.8"E	1.2	336.6	0.9	9.3	0.9	323.8	18.5	88.8	1.4	478.5	3.3	14.2	4.4
		Gahai Lake1	QSEDC1	36º43'50.4"N/100º40'19.2"E	1.5	400.0	29.0	8.6	2.6	9041.9	450.6	1168.7	0.0	12867.4	5448.1	17.0	465.1
		Gahai Lake1	QSEDC2	36º43'50.4"N/100º40'19.2"E	1.5	400.0	32.2	8.3	2.1	10069.9	488.2	1433.3	0.0	14765.7	5393.3	50.4	446.7
		Gahai Lake1	QSEDC3	36º43'50.4"N/100º40'19.2"E	1.5	400.0	28.9	8.7	1.8	9226.3	446.8	1323.6	0.0	13256.2	4604.0	47.1	453.5
		Chaka Lake	QSEDD1	36º45'01.4"N/99º04'48.1"E	3.5	159.3	363.1	7.3	0.0	67103.0	6609.5	40471.3	132.8	207796.7	40977.8	475.0	416.1
		Chaka Lake	QSEDD2	36º45'01.4"N/99º04'48.1"E	3.5	159.3	373.3	6.9	0.0	71557.0	6460.8	38959.8	135.2	209155.8	47032.2	569.6	0.0
		Chaka Lake	QSEDD3	36º45'01.4"N/99º04'48.1"E	3.5	159.3	357.6	6.9	0.0	65889.9	6441.4	39005.1	151.8	204396.0	41711.6	421.4	0.0
		Gahai Lake2	QSEDE1	37º08'10.8"N/97º34'38.2"E	3.0	100.0	71.7	8.2	4.6	21715.7	357.6	3175.9	81.6	37193.9	9137.2	52.1	0.0
		Gahai Lake2	QSEDE2	37º08'10.8"N/97º34'38.2"E	3.0	100.0	17.4	8.3	4.2	5056.6	58.2	883.2	115.7	8851.0	2409.8	0.0	0.0
		Gahai Lake2	QSEDE3	37º08'10.8"N/97º34'38.2"E	3.0	100.0	40.7	8.3	1.1	12788.9	197.6	2002.4	62.5	20959.1	4730.1	0.0	414.6

		Xiaochaidan Lake	QSEDF1	37º27'09.2"N/95º30'38.1"E	1.4	83.5	118.9	8.4	0.4	39176.5	706.9	2366.8	139.4	53103.7	23435.4	44.6	411.2
		Xiaochaidan Lake	QSEDF2	37º27'09.2"N/95º30'38.1"E	1.4	83.5	74.2	8.5	0.5	34205.6	584.3	2191.1	0.0	30262.9	6912.5	0.0	450.6
		Xiaochaidan Lake	QSEDF3	37º27'09.2"N/95º30'38.1"E	1.4	83.5	127.7	8.1	0.8	43029.4	703.3	2641.1	147.3	56853.3	24363.4	133.2	0.0
		Tuosu Lake	QSEDG1	37º11'38.9"N/96º53'19.4"E	3.0	100.0	27.2	8.9	0.7	7676.6	206.5	1833.4	0.0	12318.2	5168.1	0.0	0.0
		Tuosu Lake	QSEDG2	37º11'38.9"N/96º53'19.4"E	3.0	100.0	29.3	8.9	0.4	8071.3	202.2	1927.3	0.0	12795.5	6313.2	0.0	415.8
		Tuosu Lake	QSEDG3	37º11'38.9"N/96º53'19.4"E	3.0	100.0	28.9	9.0	0.2	7787.9	204.5	1799.2	0.0	12473.5	6594.3	15.0	482.1
	**Tibet**	Angrenjincuo	TSEDA	29º18'30.1"N/87º13'10.8"E	6.5	400.0	0.2	9.8	0.5	70.2	3.0	10.5	7.6	21.0	45.9	0.0	40.6
		Langcuo	TSEDB	29º12'44.1"N/87º22'58.8"E	6.5	400.0	0.9	9.6	0.4	707.5	13.7	103.4	0.5	34.5	41.7	0.0	0.0
		Jiadamangcuo	TSEDC	29°36'26.7"N/85°44'55.2"E	4.0	300.0	0.4	7.3	0.5	311.0	46.4	13.0	9.7	21.5	31.3	0.0	42.0
		Namucuo	TSEDG1	30°52'17.3"N/91°2'40.9"E	1.3	456.8	0.4	9.1	2.1	58.0	13.9	105.3	157.6	21.0	46.6	0.0	41.3
		Namucuo	TSEDG2	30°52'17.3"N/91°2'40.9"E	1.3	456.8	0.3	9.0	3.6	30.4	15.5	113.2	58.3	13.9	34.9	0.0	45.4
		Namucuo	TSEDG3	30°52'17.3"N/91°2'40.9"E	1.3	456.8	0.3	9.1	1.0	39.5	14.3	130.6	57.2	15.0	27.6	0.0	45.6
		Yangzhuoyongcuo	TSEDH1	29°07'36.1"N/90°27'0.4"E	3.8	353.2	0.5	8.8	2.6	197.9	20.1	116.3	93.0	18.0	79.5	0.0	40.8

		Yangzhuo yongcuo	TSEDH2	29°07'36.1"N/90°27'0.4"E	3.8	353.2	0.6	8.8	2.0	215.3	22.3	176.2	83.1	33.0	96.6	0.0	40.9
		Yangzhuo yongcuo	TSEDH3	29°07'36.1"N/90°27'0.4"E	3.8	353.2	0.7	8.8	1.7	255.6	25.2	232.2	41.4	25.7	111.9	0.0	43.3
		Bangecuo	TSEDK	31°45'05.1"N/89°30'20.1"E	0.0	340.5	57.3	9.5	2.5	22412.7	2726.4	444.0	0.0	13831.2	17845.6	462.6	36.3
	**Xinjiang**	Wulungu Lake	XSEDA1	47º13'18.0"N/87º22'32.0"E	4.7	131.0	2.2	8.6	0.0	329.2	45.3	0.0	0.0	603.8	1172.2	0.0	459.1
		Wulungu Lake	XSEDA2	47º13'14.8"N/87º22'29.2"E	4.7	131.0	2.1	8.5	0.0	338.5	39.5	46.2	0.0	577.8	1057.6	0.0	403.1
		Wulungu Lake	XSEDA3	47º13'16.8"N/87º22'26.4"E	4.7	131.0	2.0	8.6	0.2	326.1	47.0	0.0	0.0	599.1	1054.0	0.0	400.9
		Sailimu Lake	XSEDB1	44º33'13.7"N/81º20'37.4"E	1.1	400.0	2.0	8.8	6.2	209.2	10.9	265.1	82.3	424.2	1043.9	0.0	0.0
		Chaiwobu Lake	XSEDE1	43º30'29.5"N/87º56'09.3"E	6.0	70.0	16.4	7.8	4.0	6995.9	147.6	345.9	0.0	4469.8	4478.5	244.4	0.0

Table S2A Alpha-diversity of the water in the studied lakes.

Sample	Total reads	Observed_species	Simpson	Shannon	Equitability	Chao1
**QWA1**	15568	279.6	0.9	5.3	0.7	823.7
**QWA2**	7605	257.9	0.9	5.2	0.7	749.2
**QWB1**	12394	320.3	1.0	6.4	0.8	900.7
**QWB2**	8860	324.0	1.0	6.3	0.8	932.3
**QWB3**	12467	315.7	1.0	6.3	0.8	871.3
**QWC1**	6205	354.8	0.9	5.9	0.7	1081.1
**QWC2**	2731	337.0	0.9	5.7	0.7	1164.1
**QWC3**	19027	336.4	0.9	5.6	0.7	1006.3
**QWD1**	16375	240.6	0.9	5.7	0.7	522.0
**QWD2**	15239	249.4	0.9	5.8	0.7	558.5
**QWD3**	5116	216.6	0.9	5.6	0.7	440.2
**QWE1**	14193	242.4	0.9	4.7	0.6	793.8
**QWE2**	3983	287.8	0.9	5.3	0.6	966.9
**QWE3**	3988	268.5	0.9	5.1	0.6	844.8
**QWF1**	15055	233.8	0.8	4.3	0.5	862.6
**QWF2**	4152	297.9	0.9	5.3	0.6	884.7
**QWF3**	10171	397.4	1.0	6.5	0.7	1091.7
**QWG1**	3952	342.3	1.0	6.2	0.7	1104.0
**QWG2**	18140	334.1	1.0	6.2	0.7	1036.9
**QWG3**	15518	307.8	1.0	6.2	0.7	799.1
**TWA**	19011	271.7	0.9	5.2	0.6	805.0
**TWB**	6515	370.3	0.9	6.3	0.7	1049.8
**TWC**	17123	584.3	1.0	8.0	0.9	1481.0
**TWG1**	12056	369.3	0.9	6.3	0.7	1179.4

**TWG2**	6747	337.9	1.0	6.2	0.7	1019.8
**TWG3**	15591	370.4	1.0	6.4	0.7	1174.5
**TWH1**	20034	217.1	0.9	5.5	0.7	645.5
**TWH2**	3595	265.0	0.9	5.5	0.7	875.2
**TWH3**	9965	279.0	0.9	5.6	0.7	947.7
**TWK**	13952	322.8	0.9	5.4	0.6	1112.9
**XWA1**	13338	312.9	0.9	5.7	0.7	954.3
**XWA2**	4809	394.4	0.9	6.4	0.7	1131.3
**XWA3**	17774	297.4	0.9	5.8	0.7	873.9
**XWB1**	12631	242.0	1.0	5.7	0.7	916.9
**XWE1**	6889	470.4	1.0	7.0	0.8	1423.5

Table S2B Alpha-diversity of the sediment in the studied lakes.

Sample	Total reads	Observed_species	Simpson	Shannon	Equitability	Chao1
**QSEDA1**	2316	539.8	1.0	7.6	0.8	1415.0
**QSEDA2**	9779	701.5	1.0	8.6	0.9	1790.7
**QSEDB1**	4151	626.5	1.0	8.4	0.9	1599.8
**QSEDB2**	3063	648.4	1.0	8.4	0.9	1725.9
**QSEDB3**	13719	675.9	1.0	8.6	0.9	1653.5
**QSEDC1**	12248	440.3	1.0	7.2	0.8	1130.9
**QSEDC2**	11714	370.9	0.9	6.4	0.8	1042.0
**QSEDC3**	9111	467.7	1.0	7.4	0.8	1164.6
**QSEDD1**	15019	456.5	0.9	6.5	0.7	1338.7
**QSEDD2**	18068	361.1	0.9	6.1	0.7	969.6
**QSEDD3**	14443	391.6	0.9	6.3	0.7	1148.5
**QSEDE1**	5806	498.1	1.0	7.5	0.8	1281.4
**QSEDE2**	3834	612.8	1.0	8.0	0.9	1680.4
**QSEDE3**	4229	577.6	1.0	7.9	0.9	1577.6
**QSEDF1**	33620	369.6	1.0	6.7	0.8	987.1
**QSEDF2**	24945	326.5	1.0	6.5	0.8	846.0
**QSEDF3**	13627	341.2	1.0	6.4	0.8	933.0
**QSEDG1**	5339	448.1	0.9	6.1	0.7	1269.8
**QSEDG2**	12964	375.6	1.0	7.1	0.8	920.6
**QSEDG3**	5290	513.5	1.0	7.9	0.9	1278.0
**TSEDA**	10096	685.9	1.0	8.5	0.9	1716.0
**TSEDB**	6808	524.0	1.0	7.1	0.8	1486.3
**TSEDC**	12238	508.4	1.0	7.1	0.8	1445.1
**TSEDG1**	14109	648.6	1.0	8.2	0.9	1662.7

**TSEDG2**	2772	611.9	1.0	8.2	0.9	1563.3
**TSEDG3**	6185	575.9	1.0	7.8	0.9	1435.8
**TSEDH1**	11292	530.0	1.0	7.7	0.9	1422.4
**TSEDH2**	7479	780.1	1.0	8.7	0.9	2128.7
**TSEDH3**	9898	644.7	1.0	8.1	0.9	1789.9
**TSEDK**	13396	308.0	0.7	4.5	0.5	1024.7
**XSEDA1**	9662	647.3	1.0	8.2	0.9	1734.3
**XSEDA2**	1702	684.0	1.0	8.5	0.9	1832.4
**XSEDA3**	9920	499.2	1.0	7.1	0.8	1448.7
**XSEDB1**	7417	632.0	1.0	7.9	0.8	1798.5
**XSEDE1**	7038	476.6	1.0	7.6	0.8	1295.1

Table S3A Distribution and relative abundance of the abundant and rare OTUs of the water in the studied lakes.

Sample_ID	Abundant OTUs	Percentage of abundant OTUs in total OTUs within a sample	Rare OTUs	Percentage of rare OTUs in total OTUs within a sample	Relative abundance of abundant OTUs within a sample	Relative abundance of rare OTUs within a sample
**QWA1**	14	4.53	211	68.3	65.4	12.5
**QWA2**	14	5.20	182	67.7	70.3	10.8
**QWB1**	20	5.76	234	67.4	62.4	13.8
**QWB2**	17	4.84	240	68.4	57.8	14.2
**QWB3**	16	4.64	214	62.0	57.0	12.7
**QWC1**	14	3.72	268	71.3	63.6	15.9
**QWC2**	13	3.57	262	72.0	64.0	15.5
**QWC3**	10	2.94	241	70.9	64.9	14.3
**QWD1**	15	5.77	151	58.1	60.8	8.9
**QWD2**	13	4.85	142	53.0	57.1	8.4
**QWD3**	19	8.68	106	48.4	68.1	6.3
**QWE1**	12	4.56	188	71.5	74.9	11.1
**QWE2**	11	3.56	221	71.5	67.7	13.1
**QWE3**	12	4.03	215	72.2	69.8	12.7
**QWF1**	13	4.69	224	80.9	78.5	13.3
**QWF2**	11	3.20	242	70.4	64.6	14.3
**QWF3**	15	3.44	292	67.0	56.1	17.3
**QWG1**	18	5.20	244	70.5	66.1	14.4
**QWG2**	16	4.52	233	65.8	61.9	13.8
**QWG3**	18	5.56	202	62.4	63.9	12.0
**TWA**	15	5.17	191	65.9	66.8	11.3

**TWB**	17	4.12	273	66.1	56.1	16.1
**TWC**	15	2.45	396	64.7	33.7	23.4
**TWG1**	19	4.74	282	70.3	58.0	16.7
**TWG2**	16	4.43	252	69.8	58.3	14.9
**TWG3**	18	4.32	296	71.0	56.1	17.5
**TWH1**	20	8.10	163	66.0	71.5	9.6
**TWH2**	15	5.49	191	70.0	69.7	11.3
**TWH3**	17	5.84	199	68.4	69.4	11.8
**TWK**	11	3.24	242	71.2	64.5	14.3
**XWA1**	14	3.98	245	69.6	60.7	14.5
**XWA2**	13	3.19	269	65.9	51.3	15.9
**XWA3**	17	5.57	192	63.0	62.4	11.4
**XWB1**	18	6.87	177	67.6	72.3	10.5
**XWE1**	15	3.02	345	69.4	46.3	20.4
**QWA1**	14	4.53	211	68.3	65.4	12.5
**QWA2**	14	5.20	182	67.7	70.3	10.8
**QWB1**	20	5.76	234	67.4	62.4	13.8
**QWB2**	17	4.84	240	68.4	57.8	14.2
**QWB3**	16	4.64	214	62.0	57.0	12.7
**QWC1**	14	3.72	268	71.3	63.6	15.9
**QWC2**	13	3.57	262	72.0	64.0	15.5
**QWC3**	10	2.94	241	70.9	64.9	14.3
**QWD1**	15	5.77	151	58.1	60.8	8.9

Table S3B Distribution and relative abundance of the abundant and rare OTUs of the sediment in the studied lakes.

Sample_ID	Abundant OTUs	Percentage of abundant OTUs in total OTUs within a sample	Rare OTUs	Percentage of rare OTUs in total OTUs within a sample	Relative abundance of abundant OTUs within a sample	Relative abundance of rare OTUs within a sample
**QSEDA1**	12	2.07	372	64.0	33.0	22.0
**QSEDA2**	11	1.46	509	67.6	20.5	30.1
**QSEDB1**	16	2.41	414	62.3	22.2	24.5
**QSEDB2**	10	1.44	461	66.5	19.9	27.3
**QSEDB3**	7	0.95	483	65.7	16.1	28.6
**QSEDC1**	19	3.88	323	65.9	46.1	19.1
**QSEDC2**	15	3.71	268	66.3	51.1	15.9
**QSEDC3**	16	3.21	326	65.5	40.6	19.3
**QSEDD1**	9	1.81	338	68.2	45.6	20.0
**QSEDD2**	13	3.26	266	66.7	55.0	15.7
**QSEDD3**	10	2.38	276	65.6	48.6	16.3
**QSEDE1**	16	3.05	345	65.7	42.6	20.4
**QSEDE2**	14	2.16	439	67.6	31.2	26.0
**QSEDE3**	12	1.91	430	68.5	30.3	25.4
**QSEDF1**	20	4.87	267	65.0	55.2	15.8
**QSEDF2**	19	5.72	206	62.1	59.8	12.2
**QSEDF3**	17	4.70	245	67.7	60.1	14.5
**QSEDG1**	7	1.46	336	70.0	47.1	19.9
**QSEDG2**	19	4.33	286	65.2	44.5	16.9
**QSEDG3**	18	3.27	337	61.2	31.6	19.9
**TSEDA**	9	1.24	475	65.6	19.3	28.1

**TSEDB**	8	1.43	387	69.0	39.9	22.9
**TSEDC**	11	2.03	369	68.2	41.3	21.8
**TSEDG1**	11	1.60	444	64.5	25.8	26.3
**TSEDG2**	12	1.85	433	66.6	26.9	25.6
**TSEDG3**	13	2.09	407	65.4	29.6	24.1
**TSEDH1**	14	2.42	397	68.6	33.3	23.5
**TSEDH2**	6	0.73	564	68.3	15.7	33.4
**TSEDH3**	10	1.44	487	70.3	27.1	28.8
**TSEDK**	10	3.02	244	73.7	67.6	14.4
**XSEDA1**	11	1.63	449	66.6	26.6	26.6
**XSEDA2**	10	1.36	500	68.0	19.2	29.6
**XSEDA3**	11	2.12	354	68.2	44.7	20.9
**XSEDB1**	13	1.89	477	69.4	31.5	28.2
**XSEDE1**	23	4.41	350	67.2	42.1	20.7
**QSEDA1**	12	2.07	372	64.0	33.0	22.0
**QSEDA2**	11	1.46	509	67.6	20.5	30.1
**QSEDB1**	16	2.41	414	62.3	22.2	24.5
**QSEDB2**	10	1.44	461	66.5	19.9	27.3
**QSEDB3**	7	0.95	483	65.7	16.1	28.6
**QSEDC1**	19	3.88	323	65.9	46.1	19.1
**QSEDC2**	15	3.71	268	66.3	51.1	15.9
**QSEDC3**	16	3.21	326	65.5	40.6	19.3

Table S4 SIMPER analyses identify the top classes that contributed at least 1% to the dissimilarity between the microbial communities of three sampling locations.

	Class	Average dissimilarity %	Contribution %	Mean abundance % (Qinghai)	Mean abundance % (Tibet)	Mean abundance % (Xinjiang)
WATER	*Actinobacteria*	6.5	12.1	12.4	14.2	27.4
	Synechococcales	6.2	11.6	5.8	17.9	6.4
	Alphaproteobacteria	6.1	11.3	17.7	8.5	8.9
	Betaproteobacteria	5.6	10.4	5.5	12.9	17.4
	Flavobacteriia	5.5	10.3	14.2	9.2	9.1
	Halobacteria	5.3	10.0	12.5	0.3	0.4
	Cytophagia	3.9	7.3	1.3	11.5	3.6
	Saprospirae	2.6	4.9	2.0	6.0	7.5
	Rhodothermi	2.4	4.4	6.0	0.7	1.1
	Gammaproteobacteria	2.3	4.3	5.9	3.3	3.1
	Spartobacteria	1.4	2.6	2.9	0.3	1.1
	Sphingobacteriia	1.2	2.2	1.0	3.2	0.7
	Chloroplast	1.0	2.0	3.1	1.6	2.1
	Oscillatoriophycideae	1.0	1.9	0.3	2.8	0.4
	Planctomycetia	1.0	1.8	1.9	1.1	1.4
	Others	1.5	2.9	7.5	6.6	9.7
SEDIMENT	*Betaproteobacteria*	7.5	14.7	5.8	22.3	8.9
	Chloroplast	3.9	7.6	6.0	5.3	7.5
	Deltaproteobacteria	3.6	6.9	10.0	7.0	5.2
	Gammaproteobacteria	3.5	6.9	11.2	4.2	4.6
	Thaumarchaeota	2.7	5.2	2.3	1.2	10.9
	Oscillatoriophycideae	2.6	5.1	0.3	7.3	0.3

Anaerolineae	2.6	5.0	5.1	2.8	7.6
Alphaproteobacteria	2.3	4.4	8.7	4.6	6.1
Clostridia	2.2	4.2	4.4	2.0	3.4
Synechococcales	2.2	4.2	5.4	2.8	2.8
Bacteroidia	1.9	3.8	4.9	5.8	1.3
Rhodothermi	1.7	3.4	4.5	0.8	0.8
Cytophagia	1.6	3.2	1.6	3.7	5.5
Flavobacteriia	1.6	3.2	4.7	5.2	2.5
Planctomycetia	1.6	3.1	3.1	1.5	6.4
Saprospirae	1.5	2.9	2.2	3.2	5.8
Thermoplasmata	1.4	2.8	2.1	1.8	0.1
MCG	1.0	2.0	1.1	1.4	1.3
Deinococci	0.7	1.3	1.7	0.7	0.5
Halobacteria	0.6	1.2	1.7	0.4	0.4
Spirochaetes	0.6	1.2	1.4	0.9	0.1
Actinobacteria	0.4	0.9	1.7	1.7	1.5
Others	3.6	7.0	10.1	13.5	16.6

Table S5 Dissimilarity tests of the total, abundant and rare communities among the samples of different locations (i.e. Tibet, Qinghai and Xinjiang Provinces).

	All		Abundant		Rare	
Data set	R	*P*	R	*P*	R	*P*
Water	0.173	0.021	0.295	< 0.001	0.098	0.106
Sediment	0.226	0.004	0.220	< 0.001	0.106	0.087

Fig. S1

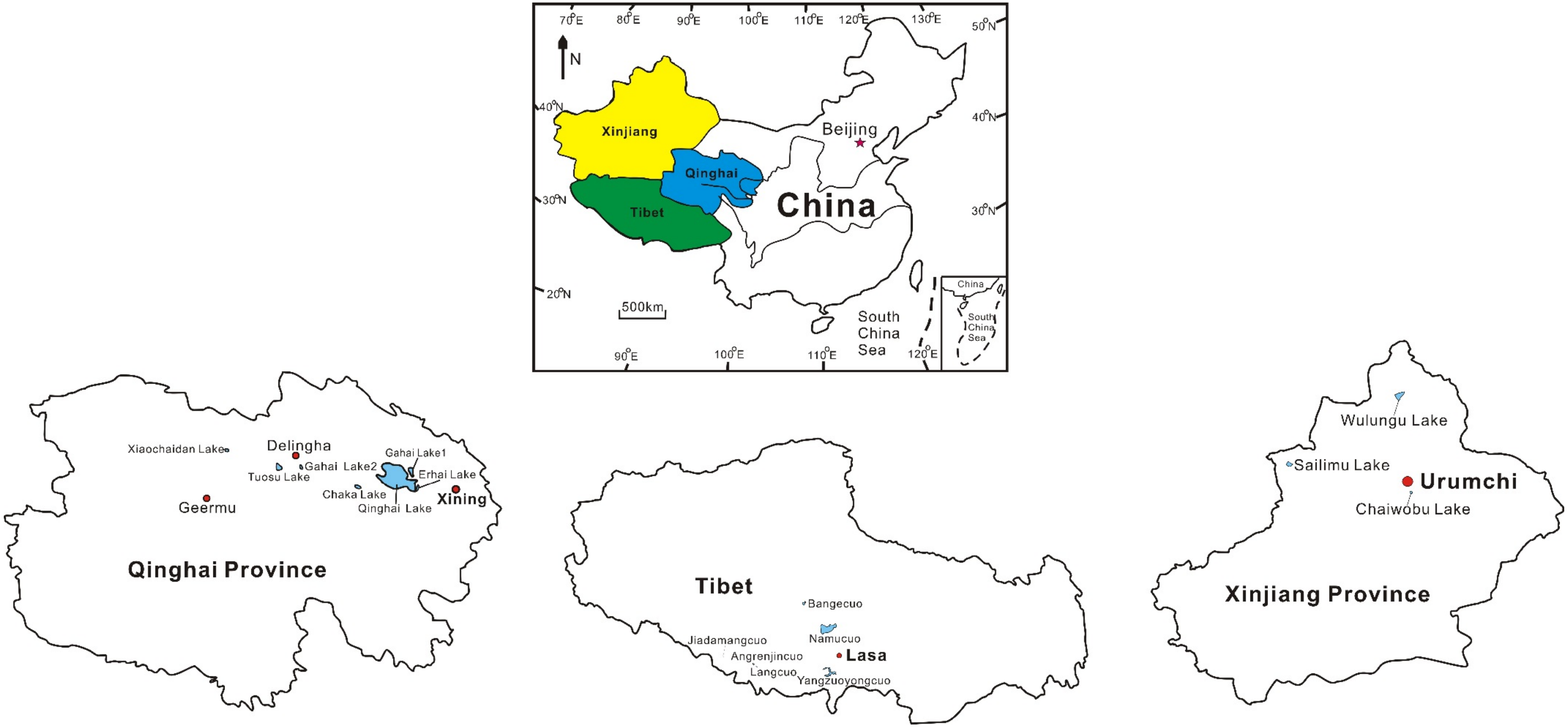
70°E
80°E
90°E
100°E
110°E
120°E
130°E
50°N
40°N
30°N
20°N
N
Xinjiang
Qinghai
Tibet
Beijing
China
500km
South
China
Sea
China
South
China
Sea
90°E
100°E
110°E
120°E
Xiaochaidan Lake
Delingha
Gahai Lake1
Gahai Lake2
Tuosu Lake
Erhai Lake
Chaka Lake
Xining
Qinghai Lake
Geermu
Qinghai Province
Tibet
Bangecuo
Namucuo
Jiadamangcuo
Angrenjincuo
Lasa
Langcuo
Yangzuoyongcuo
Wulungu Lake
Sailimu Lake
Urumchi
Chaiwobu Lake
Xinjiang Province
